# QOGMP: QoS-oriented global multi-path traffic scheduling algorithm in software defined network

**DOI:** 10.1038/s41598-022-18919-w

**Published:** 2022-08-26

**Authors:** Yiping Guo, Guyu Hu, Dongsheng Shao

**Affiliations:** 1 Command and Control Engineering College, People’s Liberation Army Engineering University, Nanjing, CO 210007 China; 2 Unit 31106 of People’s Liberation Army, Nanjing, CO 210007 China

**Keywords:** Communication and replication, Computational platforms and environments, Machine learning, Network topology

## Abstract

According to the research status of Software Defined Network (SDN) control layer traffic scheduling, we find the current common problems, including single path, easy congestion, Quality of Service (QoS) requirements and high delay. To solve these four problems, we design and implement a QoS-oriented global multi-path traffic scheduling algorithm for SDN, referred to as QOGMP. First, we propose a link weight calculation algorithm based on the idea of traction links and deep reinforcement learning, and conduct experimental verifications related to traction links. The algorithm considers QoS requirements and alleviates the problems of easy congestion and high delay. Then, we propose a traffic scheduling algorithm based on link weight and multi-path scheme, which also considers QoS requirements and solves the problem of single path. Finally, we combined the link weight calculation algorithm and the traffic scheduling algorithm to implement QOGMP, and carried out comparative experiments in the built simulation environment. The experimental results show that QOGMP is better than the two comparison algorithms in terms of delay and rescheduling rate.

## Introduction

With the continuous expansion of network scale and continuous updating of network technology, high availability and high performance requirements are put forward to the network. In order to maintain network availability and improve network performance, it is necessary to effectively allocate network resources and reasonably schedule network traffic^1^. Traffic scheduling is the process of assigning concurrent request packets of massive users to server program instances of different IP addresses according to a specific strategy.

With the continuous increase in the number and types of devices connected to the network, in the face of the rapidly changing business environment of the Internet, the drawbacks of the traditional network have gradually become apparent^2^. Under the new situation of rapid network development and continuous emergence of network applications, traffic scheduling based on the traditional network has limitations: (1) It challenges the efficiency of traffic scheduling. The development of cloud computing has made the demand for large-scale data centers more and more obvious. In the process of network traffic scheduling, network resources should be used more effectively to reduce costs and improve the overall performance of the network^3^. However, the traditional network traffic scheduling technology has problems such as low performance and high overhead when facing large-scale networks. (2) The network is prone to congestion. In traditional network traffic scheduling, only local information can be used. The network node calculates the path to the destination node based on itself. It does not have a global network view, so it is difficult to perform good traffic scheduling^4^, which makes the probability of congestion and other problems in the network continue to increase. (3) Traffic scheduling is not flexible. In the face of rapid network changes and user demand, it is very weak. After traffic scheduling according to network application requirements, if the demand changes, the configuration of the corresponding network equipment (such as routers, switches and firewalls) needs to be revised in the traditional network^5^, so as to re-schedule the traffic, which is a very tedious process.

The birth of Software Defined Network (SDN) can solve these limitations. In 2009, Professor Mckeown formally proposed SDN, a new network architecture in the literature^6^, breaking the closed mode of integration of software and hardware of traditional network equipment, and separating the control level of network equipment from the data forwarding level.

The design concept of SDN is to separate the control layer and data layer of the network while realizing programmable control, which can provide centralized management and dynamic maintenance capabilities for distributed networks^7,8^, thereby effectively solving the disadvantages of the traditional IP network in maintenance, expansion, and experimental innovation. The typical architecture of SDN is divided into three layers^9^. The top layer is the application layer, including various services and applications. The middle layer is the control layer, which is mainly responsible for processing the data resource arrangement and maintaining information such as network topology and status. The main body of the control layer is a logically centralized and programmable controller that can master global network information, which is convenient for operators and scientific researchers to manage and configure the network and deploy new protocols. The bottom layer is the data layer, which is responsible for data processing, forwarding, and status collection based on the flow table. The main body of the data layer is a lot of dumb switches (different from the traditional two-layer switches, specifically refers to the equipment used to forward data). These switches only provide simple data forwarding functions and can quickly process matched data packets to meet the increasing demand of traffic. An open unified interface (such as OpenFlow^10^) is used to interact between the control layer and the data layer. The controller issues unified standard rules to the switch through the standard interface, and the switch only needs to perform corresponding actions in accordance with standard rules.

Different from the “slice” management of the traditional network^11^, the control layer can use the global network view and dynamic rule configuration capabilities provided by SDN to perform load balancing and flexible traffic scheduling^12^. This solves the limitations of traffic scheduling based on the traditional network to a large extent, thereby maintaining network availability and improving network performance. Therefore, it is of great significance to carry out research on SDN-based control layer traffic scheduling methods.

## Research status and content

### Research status

In recent years, many scholars have devoted themselves to the study of SDN-based control-layer traffic scheduling methods. These researches are dedicated to solving different problems, including four problems such as single path, easy congestion, QoS requirements, and high latency.

1. Single path. The means to solve the single-path problem in literature^13–17^ are all multi-path transmission. For example, literature^13^ proposed an equal-cost multi-path (ECMP) scheme, which is currently widely used. However, this type of solution has two major problems: one is that the multi-path transmission scheme implemented under specific conditions lacks versatility in the SDN environment; the other is that traffic scheduling can only use local information, which is likely to cause congestion problems.
2. Easy to congest. The traffic in the network has shown explosive growth. The traditional network architecture cannot achieve flexible, fast and effective scheduling of network traffic. In addition, it is difficult to know the load status of the path. Congestion problems are prone to occur, resulting in low link utilization. The means to solve the problem of easy congestion in literature^18–23^ include processing elephant flows and using SDN global link load information. These methods introduce additional overheads such as query detection and congestion calculation, which increase the forwarding delay to a certain extent.
3. QoS requirements. When the network is overloaded or congested, QoS can ensure that important services are not affected by delay or packet loss during the transmission process^24^, while ensuring the efficient operation of the network. At present, SDN can provide QoS guarantee through mechanisms such as flow control and bandwidth reservation, but it is difficult to meet the increasing demand for QoS of business applications^25^. A real QoS-oriented traffic scheduling scheme is needed. The main means to solve the QoS problem in literature^26–29^ is to introduce user-defined constraints and comprehensively consider the link occupancy rate and the size of the business flow. These methods also introduce additional storage overhead such as packet loss rate measurement and link occupancy calculation and the delay caused by this. There is no widely used standard for user-defined constraints, and even the SDN northbound interface (between the application layer and the control layer) as the basis for its realization has not yet a unified or recognized standard. Although its research space is relatively large, its current practicability and research value are not great.
4. High latency. The solutions to the above three problems will introduce considerable delay pressure. In addition, in the face of large-scale networks, the current routing algorithms are obviously weak. It is necessary to consider an efficient forwarding path calculation algorithm to deal with it. Machine learning algorithms can usually extract traffic characteristics automatically, and do not rely on expert experience, so they are more efficient than traditional solutions in solving traffic scheduling problems. Literature^30^ proposed an SDN global multi-path traffic scheduling algorithm based on reinforcement learning. The algorithm uses the link bandwidth information provided by the SDN data layer to update the link weights, and selects k shortest paths as the forwarding paths according to the weights. Such methods based on reinforcement learning are difficult to classify complex traffic characteristics. Literature^31^ proposed an SDN traffic scheduling algorithm based on the deep neural network. Such methods are based on deep learning. Deep learning can make up for the shortcomings of reinforcement learning but requires a large amount of labeled data to train neural networks.

### Research content

Based on the above research status, in view of the four existing problems in the SDN control layer traffic scheduling research, we consider the following solutions: (1) Using the multi-path scheduling method of the ECMP scheme to solve the single path problem. (2) Using the global information provided by the centralized controller of SDN to alleviate the congestion problem. (3) Calculating traffic forwarding rules by integrating multiple link parameters (packet loss, delay, link capacity) and the size of business traffic to provide QoS guarantee. (4) Using deep reinforcement learning algorithms to alleviate the high latency problem. The forwarding path calculation problem is suitable for deep reinforcement learning, and deep reinforcement learning algorithms can overcome the deficiencies of reinforcement learning and deep learning.

QoS refers to the service capability that a network can provide for network communication tasks. For different communication tasks, QoS needs to achieve different indicators, including packet loss tolerance, delay tolerance, link capacity and other metrics^32^. In the traditional single-path transmission mode, all QoS-related network parameters can be used for link weight calculation and the optimal path can be selected. However, when we adopt multi-path transmission, we need to balance multiple paths, considering the efficiency of the path selection scheme and the success rate of scheduling. Therefore, in order to ensure the efficiency of the path selection scheme, we combine the deep reinforcement learning algorithm to use the packet loss and delay parameters for link weight calculation. In order to improve the scheduling success rate of the scheme, we use the link capacity parameter to calculate the traffic forwarding path.

**Figure 1:**
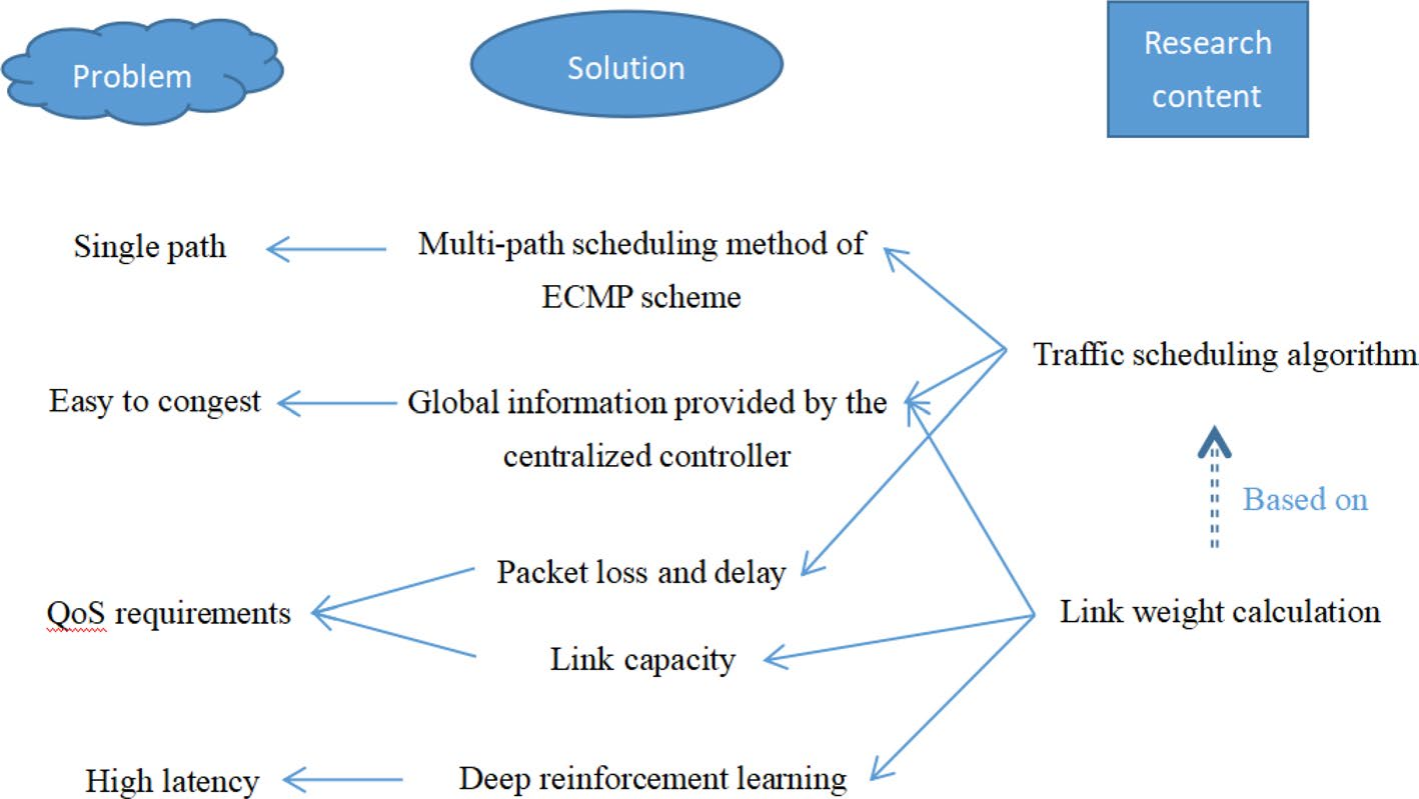
Main research content.

The main research content is as shown in Fig. 1. The paper structure is as follows: (1) In section two, we propose a link weight calculation algorithm based on the idea of traction link and deep reinforcement learning, and conduct related experiments to verify the effectiveness of traction link. This algorithm provides QoS guarantee and alleviates the problems of easy congestion and high delay. (2) In section three, we propose a traffic scheduling algorithm based on link weight and multi-path scheme, which also considers QoS requirements and solves the problem of single path. (3) In section four, we combine the algorithms proposed in section two and section three to implement a QoS-oriented global multi-path traffic scheduling algorithm for SDN, or QOGMP for short. We conduct comparative experiments in the built simulation test environment. The experimental results show that the performance of QOGMP is better than that of the algorithms for comparison.

## Link weight calculation algorithm

We design a link weight calculation algorithm based on the idea of traction link and deep reinforcement learning.

We use deep reinforcement learning algorithms to calculate link weights. The agent of the reinforcement learning system is set as a neural network (strategy generation network), and its interaction with the environment is modeled as a Markov process. The Markov process is represented by a four-tuple $$ E=<X,A,P,R>$$, where the probability *P* defaults to 1, the state of the environment $$ x\in X $$ is the current traffic view, the action $$ a\in A $$ is the link weight value, and the reward $$ r\in R $$ is the strategic value feedback provided by the environment for the neural network. By constantly trying actions, the strategy $$ \pi $$ is obtained, and the action to be executed $$ a=\pi (x) $$ can be known in the state *x*. The quality of the strategy can be measured by the value function.

The strategy generation network is implemented by the neural network $a=\pi (x|\mu )$, and its parameter is $\mu$. The strategic value network is realized by the neural network $Q(x_{t},a_{t}|\theta )$, and its parameter is $\theta$.

The value function of the strategy is expressed as Eq. (1).

1 $$\begin{aligned} Q(x_{t},a_{t})=r_{t}+\gamma Q(x_{t+1},a_{t+1}). \end{aligned}$$

The reward function of the strategy is expressed as Eq. (2).

2 $$\begin{aligned} y_{t}=r_{t}+\gamma Q(x_{t},\pi (x_{t+1})|\theta ). \end{aligned}$$

The parameters of the strategy value network and the strategy generation network need to be continuously adjusted based on the error of value and reward.

The parameter update of the strategic value network is in accordance with Eq. (3).

3 $$\begin{aligned} \triangledown _{\theta } =E[(Q(x_{t},a_{t}|\theta )-y_{t})^{2}]. \end{aligned}$$

The parameter update of the strategy generation network is in accordance with Eq. (4).

4 $$\begin{aligned} \begin{aligned} \triangledown _{\mu }&\approx E[\triangledown _{\theta }Q(x,\pi (x|\mu )|\theta )]\\ {}&=E[\triangledown _{a}Q(x,a|\theta )\triangledown _{\mu }\pi (x|\mu )]. \end{aligned} \end{aligned}$$

The input of the strategy generation network is the traffic view and reward, and the output is the link weight value we need. The traffic view is a summary of the link information collected and calculated by the data layer, including information such as nodes, links, and the cost of each link. Since different QoS requirements have different requirements for packet loss and delay, we express the link cost as Eq. (5).

5 $$\begin{aligned} cost=\alpha \cdot packetloss+\beta \cdot delay. \end{aligned}$$

In Eq. (5), $\alpha$ and $\beta$ are coefficients set according to user needs($\alpha +\beta =1$). In the following experiments, $\alpha$ and $\beta$ are both set to 0.5.

The flow of link weight calculation algorithm based on traction link and deep reinforcement learning is shown in Algoritnm 1.
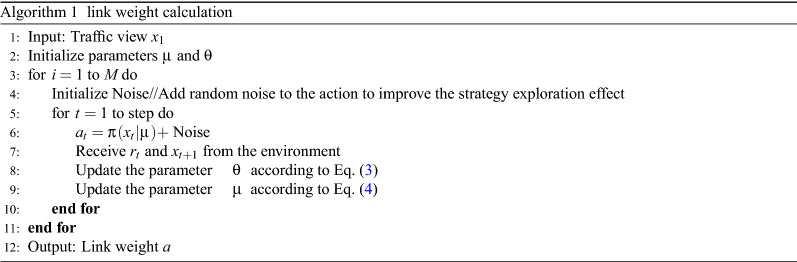


Assuming that the number of network nodes is *n*, the maximum number of optional links increases exponentially, as shown in Eq. (6).

6 $$\begin{aligned} C_{n-2}^{0}+C_{n-2}^{1}+\cdots +C_{n-2}^{n-2}=2^{n-2}. \end{aligned}$$

Although deep reinforcement learning algorithms have strong computing power, in the face of such a huge amount of data, we still need to consider reducing the number of optional links. We adopt the idea of traction link proposed in literature^33^ to alleviate this problem. Traction control theory points out that for the control of large-scale networks, it is only necessary to apply control signals to some nodes, and realize the diffusion of control signals through the connection relationship between nodes, and finally realize the coordination of the whole network, so as to achieve the control goal. For example, if there are 100 original links, the original method is to update the weight of 100 links, and finally get the path scheme L. Now, we extract 20 traction links from the original links, and update the weights of these 20 links. According to the traction control theory, the final path scheme is still L with great probability.

In the weight calculation phase, we replace original links with traction links included in original links to achieve the goal of not affecting the path selection result after the weight update, but greatly reducing the number of links to be processed.

Taking the link graph collected by the data layer as input, the flow of the traction link extraction algorithm is shown in Algoritnm 2.
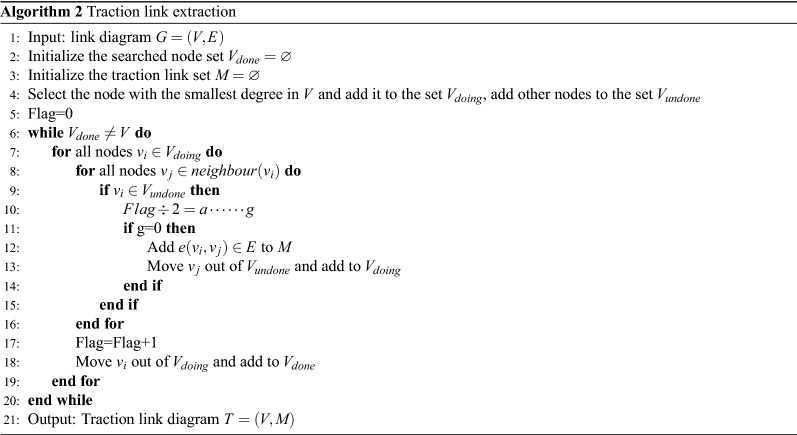


We use the traction link graph output by Algorithm 2 as the input of Algorithm 1.

## Traffic scheduling algorithm

We design a global multi-path traffic scheduling algorithm based on link weight and ECMP. Our ultimate goal is to generate a traffic scheduling scheme, that is, to calculate the traffic forwarding path and the traffic distribution on each path.

Different from the single-path scheme, the multi-path scheme may have uneven traffic distribution, resulting in low link utilization or even close to congestion, resulting in high delay and low network throughput, so that the scheduling scheme is unsuccessful and enters rescheduling. Therefore, after we update the link weight, we solve this problem by balancing the link capacity and service flow of multiple alternative paths. The link capacity and service flow data are collected by the data layer and fed back to the control layer.

How many paths do we need for traffic matching? Is it all paths? Of course not, this answer needs to be studied on the ECMP scheme to get it. The ECMP scheme is a general multi-path traffic scheduling scheme at present, which can map a single flow to multiple paths. In general, if a flow is mapped to too many paths, the delay is low but the link utilization is too low. Conversely, if a flow is mapped to too few paths, the link utilization is high but the delay is too high. We need to explore how many links a flow can be mapped to achieve the optimal compromise between link utilization and delay. To this end, we did a simple experiment.

**Table 1:** The Performance compromise record when the number of links is 3.

The number of mapped links	Transmission rate of link 1, 2, 3 (Mb/s)	Performance compromise
1	1, 2, 3	1
2	1, 2, 3	1
3	1, 2, 3	0.5
1	2, 3, 4	1
2	2, 3, 4	2.67
3	2, 3, 4	2
1	3, 4, 5	1
2	3, 4, 5	4.5
3	3, 4, 5	4.05

We assume that in a network environment, the service flow is fixed at 1 Mb and evenly distributed, and the delay is only the transmission delay. All links between the source address and the destination address are black boxes, and only the transmission rate and number of the link are known. We define a standard for the compromise between link occupancy and delay. The value of the performance compromise is equal to the product of each link occupancy divided by the longest delay in all links. The larger the value, the better the result. The definition of each link occupancy is the link delay divided by the longest delay in all links. The definition of the link delay is the service traffic allocated to the link divided by the transmission rate of the link.

When the number of links is 3, the performance compromise is calculated for different link mapping schemes and transmission rates, and the results are shown in Table 1. The number of mapped links is n, that is, the first n links are selected to calculate the performance compromise.

It can be drawn from Table 1 that the number of mapped links with the best performance compromise is 2 when the number of links is 3.

The above experimental process is a calculation from the original number of links to the number of mapped links with the best performance compromise. We perform similar calculations and records for different numbers of links, and summarize the number of mapped links with the best performance compromise, as shown in Table 2.

**Table 2:** This is a record table, where n is the number of mapped links and b is the number of mapped links with the best performance compromise.

n	b	n	b	n	b	n	b	n	b	n	b	n	b	n	b	n	b	n	b
3	2	4	2	5	2	6	2	7	3	8	3	9	3	10	3	11	3	12	3
14	4	16	4	18	4	20	4	22	5	24	5	26	5	28	5	30	5	32	6
35	6	38	6	41	6	44	7	47	6	50	7	53	7	56	8	59	8	62	8

It can be obtained from Table 2 that the number of mapped links with the best performance compromise is $[\sqrt{n}]$ when the number of mapped links is *n*. Therefore, we take $[\sqrt{n}]$ paths for traffic distribution.

There are two traffic ratio schemes: the first is to configure the ratio according to the weight on the premise that the link capacity reaches a certain requirement (to ensure that the delay is acceptable). The second is to simply allocate according to the margin ratio. The definition of the margin ratio is the capacity of each path to be allocated divided by the total capacity of all paths to be allocated. The capacity of each path is the minimum value of the capacity of all links on the path.
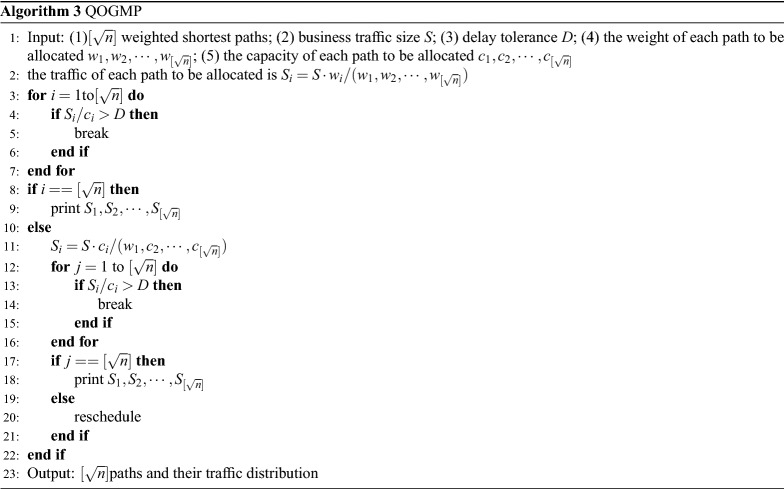


We update the link weight according to the output of Algorithm 1. If the weight is not updated, the default is 1. After calculating the $[\sqrt{n}]$ weighted shortest paths iteratively using Dijkstra’s algorithm (execute Dijkstra algorithm to find the first shortest path from the source point to the destination point, remove the first one and execute the algorithm again to find the second shortest path, and iterate until $[\sqrt{n}]$ paths are found), we execute Algorithm 3 to generate a traffic scheduling scheme.

## Simulation implementation and experiment

### Simulation implementation

Pycharm can not only run python algorithms, but also create graphical interfaces. We use the editor pycharm-community-2019.1.1 to implement the algorithm and create a simulation environment.

We combine the algorithms in section two and section three to implement the complete flow scheduling algorithm QOGMP, which is implemented in the order of Algorithm 2, Algorithm 1, and Algorithm 3. We perform a simplified simulation of the SDN network system.

The simplified network system is divided into two layers: the control layer and the data layer. The controller of the control layer has the function of receiving information from the data layer and the calculation function of the traffic scheduling scheme. The data layer has the function of data collection and flow forwarding. Data transmission is allowed between the two layers.

In the order of execution, the specific functional design ideas are as follows: (1) Data collection function of the data layer: The data layer collects network information, including switch *V*, link *E* and link parameters (cost *C*, link capacity *W*). (2) The function of the controller to receive information from the data layer: The controller obtains the traffic view *G*(*V*, *E*, *C*, *W*) fed back from the data layer. (3) The function of the controller to calculate the flow scheduling scheme: We embed the traffic scheduling algorithm into the controller as the main algorithm of the controller. We take the traffic views *G*1(*V*, *E*, *C*) , *G*2(*V*, *E*, *W*) and user requirements (including business flow and delay tolerance) as input. After the main algorithm is executed, the traffic scheduling scheme is output. (4) Flow forwarding function of the data layer: The data layer receives the scheme generated by the controller and forwards the flow according to it (the stage task is to calculate the transmission delay).

### Verification experiment of traction link

We conduct related experiments to verify the effectiveness of traction link. After implementing Algorithm 2, we record the amount of calculation saved after applying the traction link algorithm to verify whether the extraction of the traction link can greatly reduce the number of links.

**Table 3:** The extraction result of traction link.

Data set	The number of original links	The number of traction links
1	4	2
2	11	3
3	20	5
4	20	5
5	31	7
6	40	11
7	54	15
8	56	19
9	57	18
10	60	22
11	62	22
12	66	22
13	67	27
14	70	26
15	80	29
16	92	34
17	92	33
18	97	38
19	99	37
20	100	37

**Figure 2:**
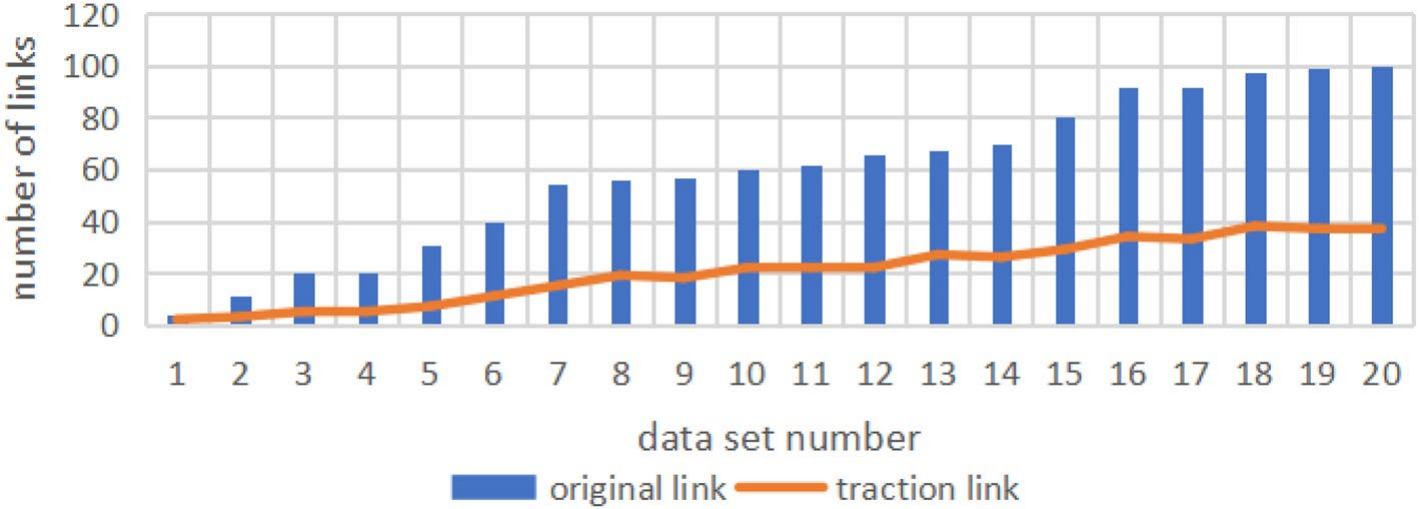
The extraction result of traction link.

We conduct experiments on 20 link graphs $G=(V, E)$ with different link numbers. Assuming that there are *n* nodes in *V*, the input format of the link graph (that is, the content of the data set) is a $n\times n$ numerical matrix, $e(i,j)=1$ indicates that there is a link between node *i* and node *j*, $e(i,j)=0$ means that there is no link between node *i* and node *j*. The data used in the experiment is randomly generated. We record the number of links in the input and output link graphs (the links between the same nodes are not recorded repeatedly), and the results are shown in Table 3 and Fig. 2.

It can be obtained from Table 3 and Fig. 2: After the application of the traction link algorithm, the saved calculation amount is up to 77 percent of the original data amount, at least 50 percent of the original data amount, and the average saved amount reaches 66 percent.

Therefore, the use of the traction link algorithm can greatly reduce the number of links that need to be processed without affecting the subsequent path selection results. It can save a large amount of calculation and improve the efficiency of the link weight calculation algorithm.

### Comparative experiment

At present, the traditional method with the best performance (delay and rescheduling rate) is the QoS-oriented SDN global traffic scheduling algorithm proposed in literature^28,29^. The machine learning algorithm with the smallest delay is the SDN global multi-path traffic scheduling algorithm based on reinforcement learning proposed in literature^30^.

**Table 4:** The comparative experiment result-delay.

Data set	Delay(s)		
	QOGMP	GMPRL	Pre-QOGMP
1	62	63	79
2	70	70	83
3	62	66	90
4	69	66	95
5	81	81	97
6	79	71	107
7	87	81	125
8	92	90	110
9	95	95	121
10	106	103	127
11	110	98	134
12	110	102	140
13	103	111	153
14	106	110	166
15	122	122	182
16	122	128	198
17	145	137	225
18	164	160	242
19	182	168	266
20	190	184	276

Compared with the algorithm proposed in literature^28,29^, QOGMP algorithm considers multi-path scheduling and has a higher link utilization rate. It uses machine learning algorithms to speed up the calculation of weights, which is suitable for big data environments. In contrast, QOGMP algorithm has obvious advantages, so it is no longer verified by comparison experiments.

We evaluate the performance of QOGMP on the built simulation system. Indicators for performance evaluation include delay and rescheduling rate. We compare QOGMP with the traffic scheduling algorithm that does not use traction links (that is, implemented in the order of Algorithm 1 and Algorithm 3, hereinafter referred to as pre-QOGMP) and the algorithm proposed literature^30^ (hereinafter referred to as GMPRL).

Delay here refers to the algorithm running time. We carry out comparative experiments on the three algorithms. In order to reduce the experimental error, each experiment needs to be measured multiple times to record the shortest running time.

For the delay indicator, the comparative experiment is completed on 20 different traffic views, and the experimental result is shown in Table 4.

**Figure 3:**
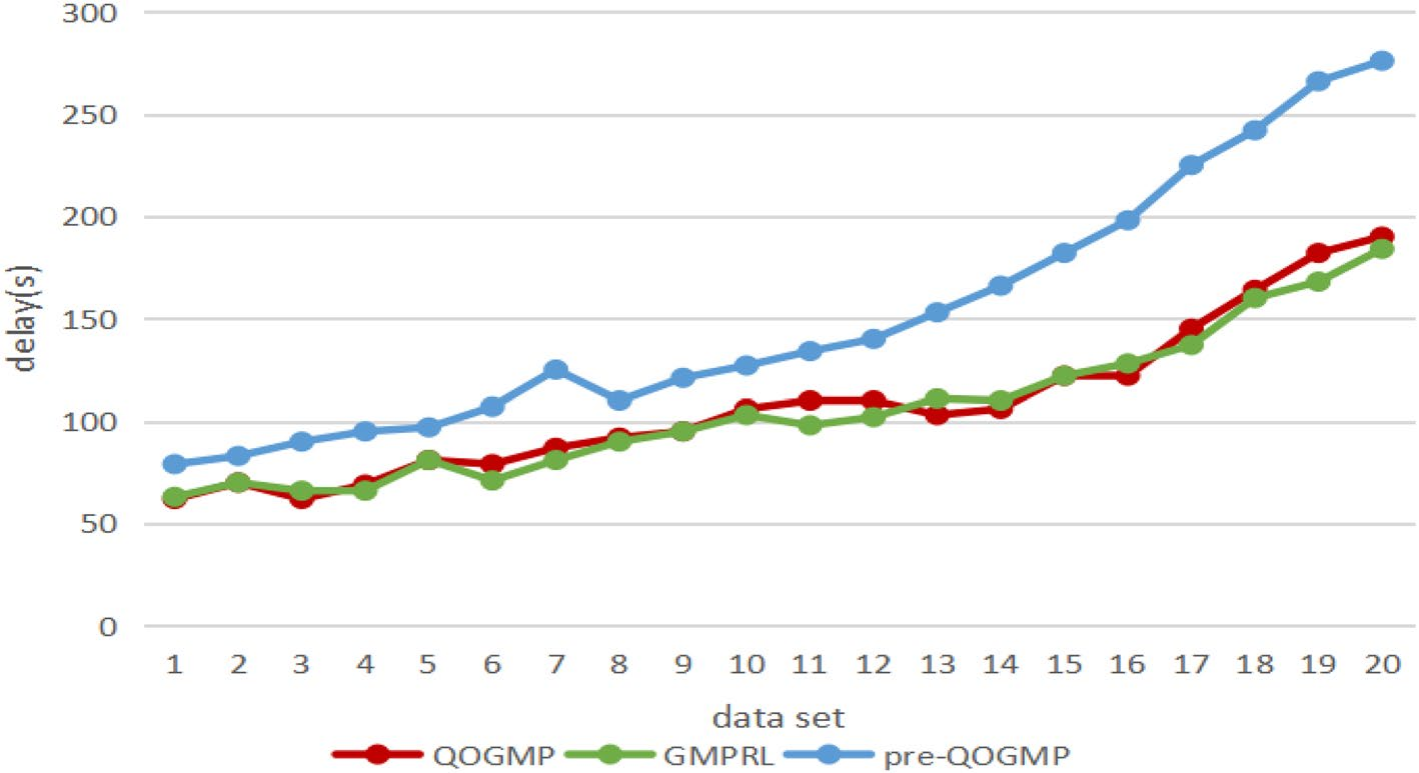
The comparative experiment result-delay.

Plot Table 4 as Fig. 3. Analysis of Fig. 3 shows that: (1) the delay of QOGMP is always lower than that of pre-QOGMP; (2) the delay of QOGMP is not much different from that of GMPRL.

**Figure 4:**
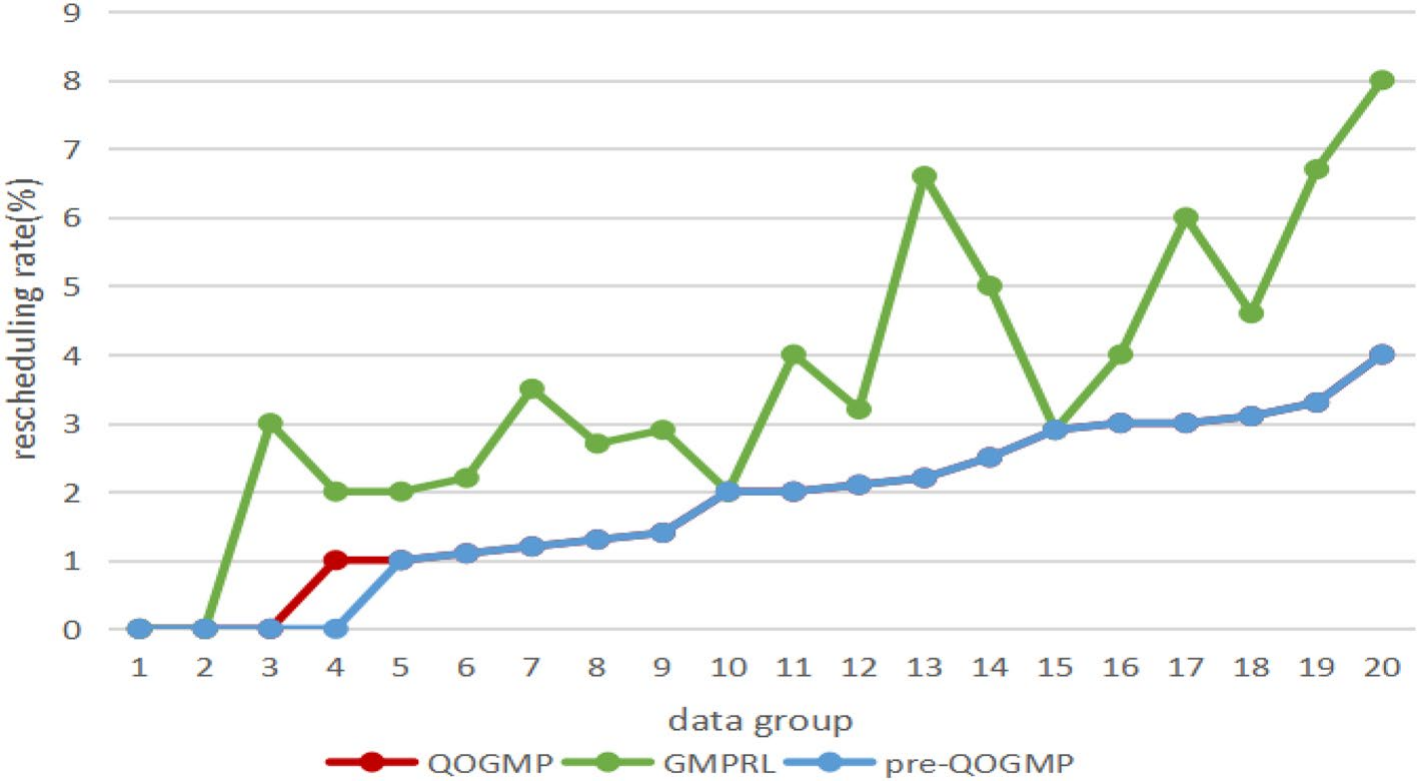
The comparative experiment result-rescheduling rate.

It can be seen from the experimental results that QOGMP is better than pre-QOGMP and is almost consistent with GMPRL in terms of delay indicator. (1) Since both QOGMP and GMPRL use machine learning algorithms, the delay of QOGMP is not much different from that of GMPRL; (2) QOGMP increases the step of pulling link extraction which leads to the increase of running time. But at the same time, the introduction of traction link greatly saves the amount of computation. So QOGMP outperforms pre-QOGMP in delay.

Since the three algorithms are all multi-path algorithms, the criteria used for rescheduling judgment are the same, that is, the sum of all path traffic in the solution generated by the algorithm is less than the service traffic or the transmission delay of a single path exceeds the delay tolerance.

For the indicator of rescheduling rate, we conduct 20 groups of comparative experiments, each of which was completed on 20–100 different data sets. The experimental result is shown in Table 5.

**Table 5:** The comparative experiment result-rescheduling rate.

Data group	Rescheduling rate (percent)		
	QOGMP	GMPRL	pre-QOGMP
1	0	0	0
2	0	0	0
3	0	3	0
4	1	2	0
5	1	2	1
6	1.1	2.2	1.1
7	1.2	3.5	1.2
8	1.3	2.7	1.3
9	1.4	2.9	1.4
10	2	2	2
11	2	4	2
12	2.1	3.2	2.1
13	2.2	6.6	2.2
14	2.5	5	2.5
15	2.9	2.9	2.9
16	3	4	3
17	3	6	3
18	3.1	4.6	3.1
19	3.3	6.7	3.3
20	4	8	4

Plot Table 5 as Fig. 4. Analysis of Fig. 4 shows that: (1) The rescheduling rate of QOGMP is always not higher than GMPRL. In 17/20 cases, the rescheduling rate of QOGMP is lower than that of GMPRL, and in 3/20 cases, the rescheduling rate of QOGMP is the same as that of GMPRL. (2) The rescheduling rate of QOGMP is almost the same as that of pre-QOGMP, and the rescheduling rate of QOGMP is slightly higher than that of pre-QOGMP only in 1/20 cases.

It can be seen from the experimental results that QOGMP is better than GMPRL and is almost consistent with pre-QOGMP in terms of rescheduling rate indicator. (1) GMPRL does not consider QoS requirements, resulting in an increased probability that the traffic scheduling scheme is not suitable for service traffic, so QOGMP outperforms GMPRL in re-scheduling rate; (2) Since the introduction of traction links will not have a great impact on the final scheme, so QOGMP is almost identical to pre-QOGMP in rescheduling rate.

To sum up, compared with pre-QOGMP, QOGMP has lower delay and almost the same rescheduling rate; compared with GMPRL, QOGMP has lower rescheduling rate and almost the same delay. Therefore, our proposed QOGMP is better than GMPRL and pre-QOGMP for delay and rescheduling rate indicators.

## Conclusion

We design and implement a QoS-oriented SDN global multi-path traffic scheduling algorithm, referred to as QOGMP. Aiming at the four problems currently existing in the research of SDN control layer traffic scheduling, QOGMP has adopted solutions: (1) QoS problem is solved by using the three QoS-related network parameters of packet loss, delay, and link capacity to generate traffic scheduling scheme; (2) The easy congestion problem is alleviated by utilizing the controller global view; (3) The high latency problem is alleviated by introducing traction links and deep reinforcement learning; (4) The single path problem is solved by multi-path scheduling. We carry out comparative experiments in the built simulation environment. The experimental results show that QOGMP has better performance than the two comparison algorithms in terms of delay and rescheduling rate.

However, we still have room for improvement in terms of application scenario expansion, application algorithm improvement, and system details reproduction. The details are as follows.

1. We only considered the three common QoS parameters of delay, packet loss and link capacity. However, the complex network environment also contains other QoS parameters such as jitter. We can conduct in-depth research on QoS parameters and improve the algorithm proposed in this article in order to further cope with the complex network environment and business requirements.
2. As the neural network is not the innovative point and focus of the QOGMP algorithm, the neural network used in this article is the most basic. It can be replaced with an improved neural network. For example, in order to improve the update stability of neural network, we can use the target network method proposed in literature^34^.
3. The delay in the comparative experiment is up to 276 s, which is caused by hardware limitations. Later, the delay can be shortened to meet practical application requirements through Brax accelerator hardware^35^.

## Data Availability

All data generated or analysed during this study are included in this published article.
